# Refining surgical techniques for efficient posterior semicircular canal gene delivery in the adult mammalian inner ear with minimal hearing loss

**DOI:** 10.1038/s41598-021-98412-y

**Published:** 2021-09-22

**Authors:** Jianliang Zhu, Jin Woong Choi, Yasuko Ishibashi, Kevin Isgrig, Mhamed Grati, Jean Bennett, Wade Chien

**Affiliations:** 1 Inner Ear Gene Therapy Program, National Institute On Deafness and Other Communication Disorders (NIDCD), National Institutes of Health, Bethesda, MD USA; 2 Department of Otorhinolaryngology-Head and Neck Surgery, Chungnam National University, College of Medicine, Daejeon, South Korea; 3 Center for Advanced Retinal and Ocular Therapeutics, University of Pennsylvania Perelman School of Medicine, Philadelphia, PA USA; 4 Department of Otolaryngology-Head and Neck Surgery, Johns Hopkins School of Medicine, Baltimore, MD USA

**Keywords:** Translational research, Sensory systems

## Abstract

Hearing loss is a common disability affecting the world’s population today. While several studies have shown that inner ear gene therapy can be successfully applied to mouse models of hereditary hearing loss to improve hearing, most of these studies rely on inner ear gene delivery in the neonatal age, when mouse inner ear has not fully developed. However, the human inner ear is fully developed at birth. Therefore, in order for inner ear gene therapy to be successfully applied in patients with hearing loss, one must demonstrate that gene delivery can be safely and reliably performed in the mature mammalian inner ear. In this study, we examine the steps involved in posterior semicircular canal gene delivery in the adult mouse inner ear. We find that the duration of perilymphatic leakage and injection rate have a significant effect on the post-surgical hearing outcome. Our results show that although AAV2.7m8 has a lower hair cell transduction rate in adult mice compared to neonatal mice at equivalent viral load, AAV2.7m8 is capable of transducing the adult mouse inner and outer hair cells with high efficiency in a dose-dependent manner.

## Introduction

Hearing loss is a common disease process affecting the world’s population today. Approximately 3 in every 1000 newborns are affected by hearing loss every year^1^. Over the past few years, several studies have shown that inner ear gene therapy is effective at improving the auditory function in mouse models of hereditary hearing loss^2^. In most of these studies, gene delivery is done in the neonatal age (< P5). One major difference between human and mouse ears is the fact that the auditory system is fully mature at birth in humans, whereas the onset of hearing is not until ~ P12 in mice^3^. The mouse inner ear is immature at birth and continues to undergo development after birth^3,4^. Therefore, in order for inner ear gene therapy to be successfully translated to patients with hereditary hearing loss, one needs to demonstrate that gene therapy can be effective when delivered to the mature mammalian inner ear. In addition, one also needs to identify viral vectors which can successfully transduce target cells in the mature mammalian inner ear.

Various gene delivery methods have been examined for delivering gene therapy to the inner ear in animal models of hearing loss^5^. Three delivery methods (cochleostomy, round window injection, and canalostomy) are commonly used in neonatal and adult mice^6^. Cochleostomy allows for transgene delivery directly into the scala media, where the mechanosensory hair cells in the cochlea are located. Even though one study showed no hearing loss in adult mice with this approach^7^, other studies have shown that this surgical approach causes significant hearing loss, likely due to the trauma incited by drilling through the lateral wall of the cochlea^8–10^. Round window injection is another method for administering gene therapy into the inner ear. The round window is a membranous structure at the base of the cochlea which separates the middle ear and the cochlea. It can be accessed via the middle ear after the opening of the tympanic bulla. Despite being less invasive than cochleostomy, it can lead to middle ear effusion, which negatively affects hearing temporarily^11^. In addition, the transduction efficiency of round window injection may not be evenly distributed throughout the cochlear turns, with a lower transduction rate in the apical turn, which is further away from the injection site (round window)^12,13^.

The canalostomy approach involves gene delivery through one of the semicircular canals located superficially in the temporal bone. It does not require opening the tympanic bulla, which minimized the chances for surgical trauma and middle ear effusion^14–16^. In rodents, the posterior semicircular canal (PSC) is the most prominent and easily accessible out of the three semicircular canals. Therefore, PSC approach is a commonly used surgical method for inner ear gene therapy studies in neonatal mice^17,18^. However, since the mouse otic capsule is initially cartilaginous and becomes ossified by postnatal week 2^4,19^, this potentially makes PSC gene delivery in adult mouse inner ear more challenging technically. In a study by Suzuki et al., they reported that the PSC approach can be successfully performed in adult mice. However, the surgical techniques for accessing the PSC and for gene delivery are significantly different than in neonatal mouse ears^20^.

In this study, we examine the surgical steps involved in the PSC approach in order to refine this surgical technique for safe and reliable gene delivery in the adult mouse inner ear. We find that the adult mouse inner ear is more susceptible to surgical trauma compared to the neonatal mouse inner ear. In addition, we find that the duration of perilymphatic leakage and injection rate have significant effect on hearing in the adult mouse inner ear. We also show that the synthetic AAV2.7m8 is capable of transducing the adult mouse inner and outer hair cells with high efficiency.

## Results

### Adult mouse inner ear is more susceptible to hearing loss than neonatal inner ear for inner ear gene delivery

**Figure 1:**
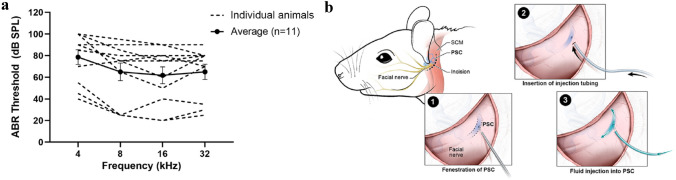
Adult mouse inner ear is more susceptible to surgical trauma with inner ear gene delivery. (**a**) ABR thresholds in adult mice that underwent PSC gene delivery. Many mice exhibited significant ABR threshold elevation, while others did not. (**b**) Schematic drawing of PSC approach used in this study for inner ear gene delivery in adult mouse. Drawing created by Alan Hoofring.

Posterior semicircular canal approach (PSC) has been shown to be a safe and effective surgical approach for inner ear gene delivery in the neonatal mouse inner ear^17,21^. In addition, some studies have also shown that it can be safely implemented in the adult mouse inner ear^15,20^. However, our initial attempts at using the PSC approach for gene delivery in the adult mouse inner ear showed significant ABR threshold elevation in many mice (Fig. 1a). Therefore, we decided to investigate the implementation of PSC approach in adult mice more thoroughly. The three main surgical steps in the PSC approach are, (1) fenestration of PSC, (2) insertion of injection tubing into the PSC, and (3) injection of fluid into the PSC (Fig. 1b). We decided to examine each of these surgical steps involved with PSC gene delivery to see if we could refine this surgical technique to minimize trauma to the adult mouse inner ear.

### The duration of perilymphatic leakage after PSC fenestration negatively affects hearing in adult mouse inner ear

**Figure 2:**
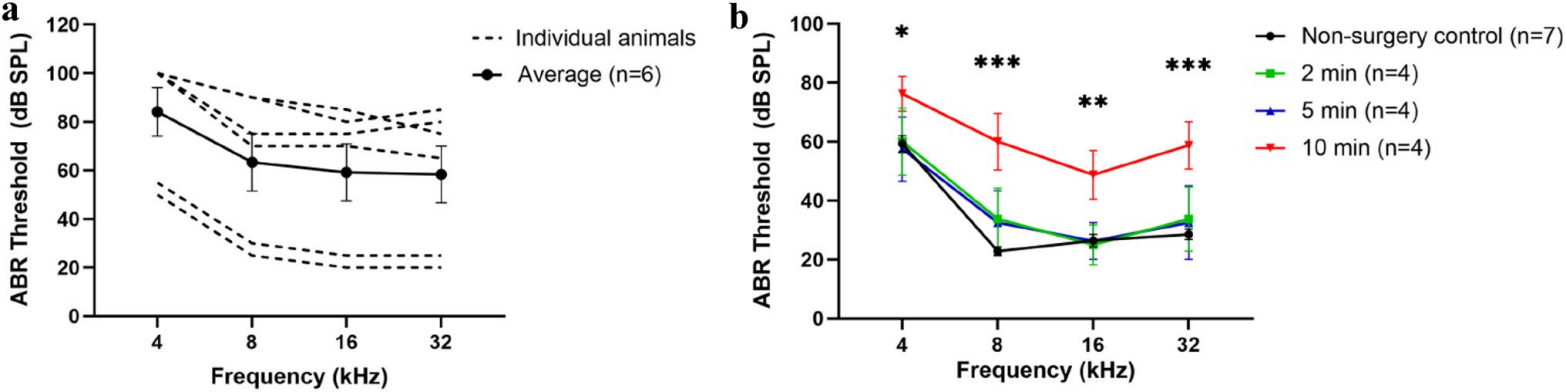
Prolonged perilymphatic leakage causes hearing loss. (**a**) ABR thresholds of adult mice undergoing PSC fenestration. Some mice developed hearing loss after PSC opening while others did not. (**b**) ABR thresholds of adult mice undergoing PSC fenestration, controlling for perilymphatic leakage time. Mice with 10-min PSC opening time exhibited significant ABR threshold elevations across all frequencies compared with non-surgery control mice (one-way ANOVA). Data are represented as mean ± SEM. n represents the number of animals tested. Statistical significance between groups is shown above error bars (*represents *p* < 0.05, **represents *p* < 0.01, ***represents *p* < 0.001).

We first examined the effect of PSC fenestration on the adult mouse inner ear. The fenestration of PSC is performed using a small 27-gauge needle to expose the canal lumen. Observation of perilymphatic leakage is used as confirmation for successful access to the PSC lumen. In this experiment, we performed PSC fenestration on adult CBA/J mice. The PSC fenestration was left open for several minutes to allow for perilymphatic leakage to occur, and then sealed off using a muscle plug. We found that 4 mice developed significant hearing loss after PSC fenestration while 2 mice did not (Fig. 2a). We decided to examine the PSC fenestration more closely by timing the duration of PSC opening and perilymphatic leakage using a new group of mice. We separated our animals into three groups based on various durations of PSC opening and perilymphatic leakage: 2, 5, and 10 min. All mice showed continuous perilymph leakage during the entire duration in which the PSC fenestration was open, though the leakage rate may decrease over time. We found that mice with 2-min and 5-min PSC opening had minimal ABR threshold elevation compared with non-surgery control mice (*p* = 0.93 and *p* = 0.97, respectively, one-way ANOVA with post-hoc Scheffe comparison). However, mice in the 10-min PSC opening group had significant ABR threshold elevation compared to the non-surgery control mice (Fig. 2b; *p* = 0.001, one-way ANOVA with post-hoc Scheffe comparison; *p* = 0.0153 for 4 kHz, *p* = 0.0006 for 8 kHz, *p* = 0.0082 for 16 kHz, and *p* = 0.00097 for 32 kHz, *t* test). Examination of the cochlea revealed significant IHC and OHC loss in animals in the 10-min PSC opening group, particularly at the cochlear base (Supplemental Fig. 1). This indicates that prolonged PSC opening time and perilymphatic leakage can adversely affect the hearing outcome in adult mouse inner ear.

### Tube insertion into the PSC had no effect on hearing in the adult mouse inner ear

**Figure 3:**
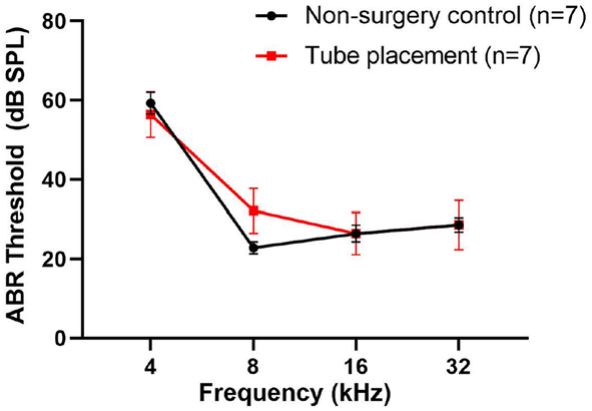
Injection tube insertion into the PSC lumen does not cause hearing loss. ABR thresholds in mice that underwent injection tube insertion are comparable to non-surgery control mice (*p* > 0.05, one-way ANOVA). Data are represented as mean ± SEM. n represents the number of animals tested.

Next, we compared ABR thresholds in mice with or without insertion of injection tubing into the PSC to determine whether this surgical step would adversely affect auditory function. It is important to remember that mice undergoing tube insertion will have to undergo PSC fenestration. Therefore, this surgical step cannot be evaluated on its own, but must be evaluated after the PSC fenestration has been created. The PSC opening time was kept below 5 min to minimize perilymphatic leakage. We found that there was no significant difference in ABR thresholds between mice that underwent tube insertion and non-surgery control mice when the PSC opening was kept below 5 min (*p* = 0.73, one-way ANOVA, Fig. 3).

### The rate of injection has significant effect on hearing in the adult mouse inner ear

**Figure 4:**
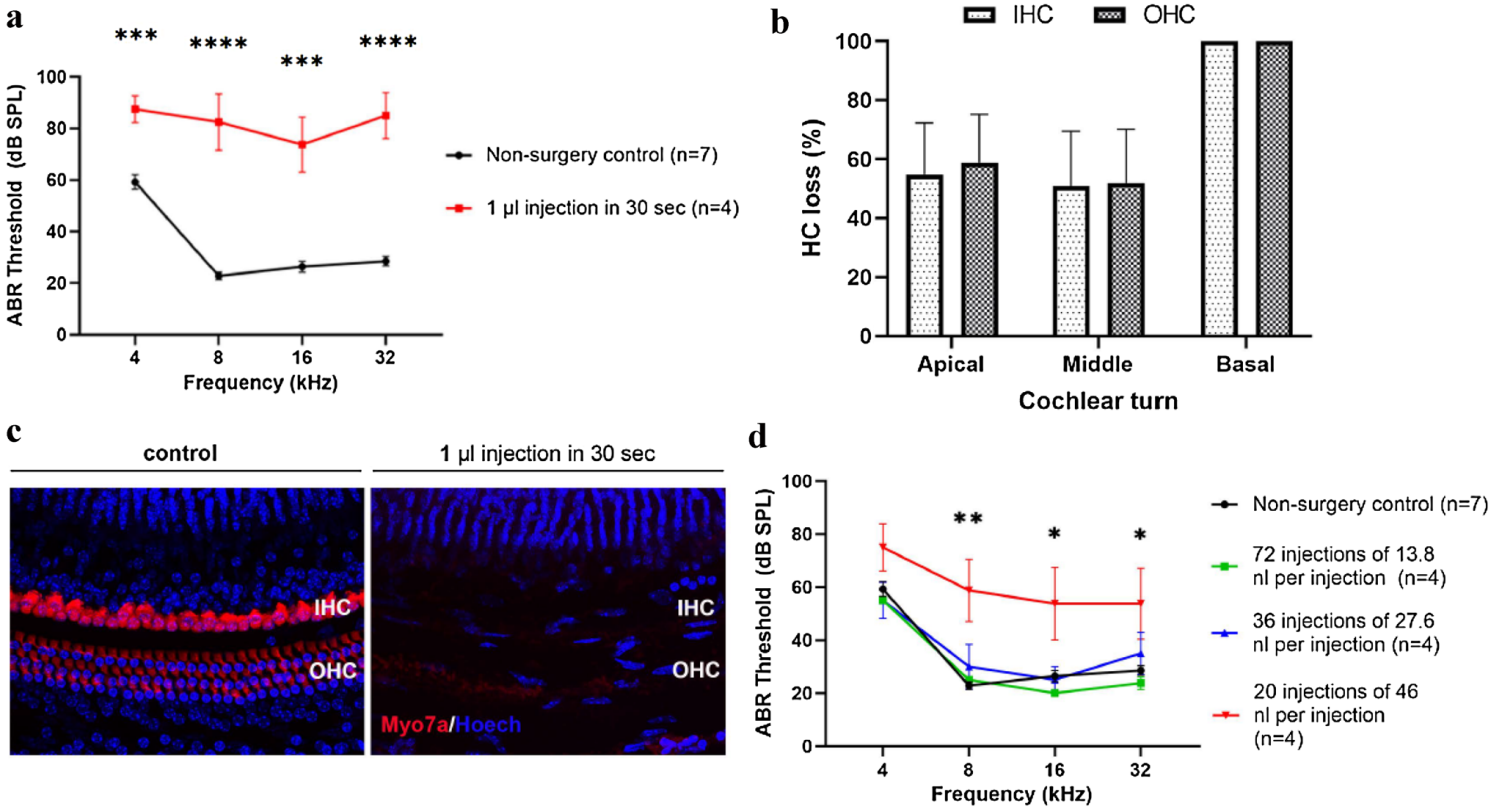
Injection rate has significant effect on hearing in adult mouse inner ear. (**a**) Adult mice that received 1 µl gene delivery within 30 s had significant ABR threshold elevation at all frequencies tested in comparison with non-injection control mice (*p* < 0.05, one-way ANOVA). (**b**) Substantial IHC and OHC losses were observed throughout the cochlear turns in mice that received 1 µl gene delivery within 30 s. (**c**) Confocal images of the basal turn of the cochlea in a non-surgery control mouse (labeled “control”) and a mouse that underwent 1 µl gene delivery within 30 s (labeled “1 µl gene delivery in 30 s”). The cochlear specimen from the animal that underwent 1 µl gene delivery within 30 s had complete loss of IHCs and OHCs. (**d**) Adult mice that underwent 20 injections of 46 nl per injection every 10 s exhibited significant ABR threshold elevation, whereas mice that received 72 injections of 13.8 nl per injection every 10 s, and 36 injections of 27.6 nl per injection every 10 s did not, compared with non-surgery control mice (one-way ANOVA). Data are represented as mean ± SEM. n represents the number of animals tested. Scale bar represents 20 μm. Statistical significance between groups is shown above error bars (*represents *p* < 0.05, **represents *p* < 0.01, ***represents *p* < 0.001, ****represents *p* < 0.0001).

Lastly, we evaluated the effect of injection rate in the adult mouse inner ear via the PSC. Again, it is important to remember that in order for mice to be injected with gene therapy, the PSC must be fenestrated first, and then the injection tubing must be inserted into the PSC lumen in order for the injection to take place. In neonatal mice, we have shown previously that we could deliver approximately 1 µl of fluid volume within a span of 30 s into the inner ear without any ABR threshold elevation compared to non-surgery control mice^18^. However, when we used the same injection rate in adult mice, significant ABR threshold elevation was observed (*p* < 0.0001, one-way ANOVA, Fig. 4a). Substantial IHC and OHC loss was found throughout the cochlear turns (Fig. 4b). In the basal turn of the cochlea, all IHCs and OHCs were damaged, suggesting the hearing loss observed resulted from hair cell damage after injection (Fig. 4c).

Therefore, we decided to examine the effect of injection rate in the adult mouse inner ear more thoroughly. The micro-injector that we use allows us to set the fluid volume per injection (e.g. 13.8 nl, 27.6 nl, 46 nl, etc.), and the injection interval can be spaced out as determined by the investigators. We assessed the following three different injection regimens: 72 injections of 13.8 nl per injection every 10 s (for a total volume of 993.6 nl), 36 injections of 27.6 nl per injection every 10 s (for a total volume of 993.6 nl), and 20 injections of 46 nl per injection every 10 s (for a total volume of 920 nl). We found that there was no significant difference in the average ABR thresholds between mice in the 13.8 nl per injection and 27.6 nl per injection groups compared to non-surgery control mice (*p* = 0.95 and 0.99, respectively, one-way ANOVA with post-hoc Scheffe comparison, Fig. 4d). However, mice in the 46 nl per injection group exhibited significantly higher ABR thresholds compared to non-surgery control mice (*p* < 0.001, one-way ANOVA with post-hoc Scheffe comparison, Fig. 4d). The differences in ABR threshold were significant at all tested frequencies except 4 kHz (*p* = 0.064 for 4 kHz, *p* = 0.0027 for 8 kHz, *p* = 0.026 for 16 kHz, and *p* = 0.032 for 32 kHz, *t* test).

### AAV2.7m8 transduced adult cochlear hair cells with high efficiency

**Figure 5:**
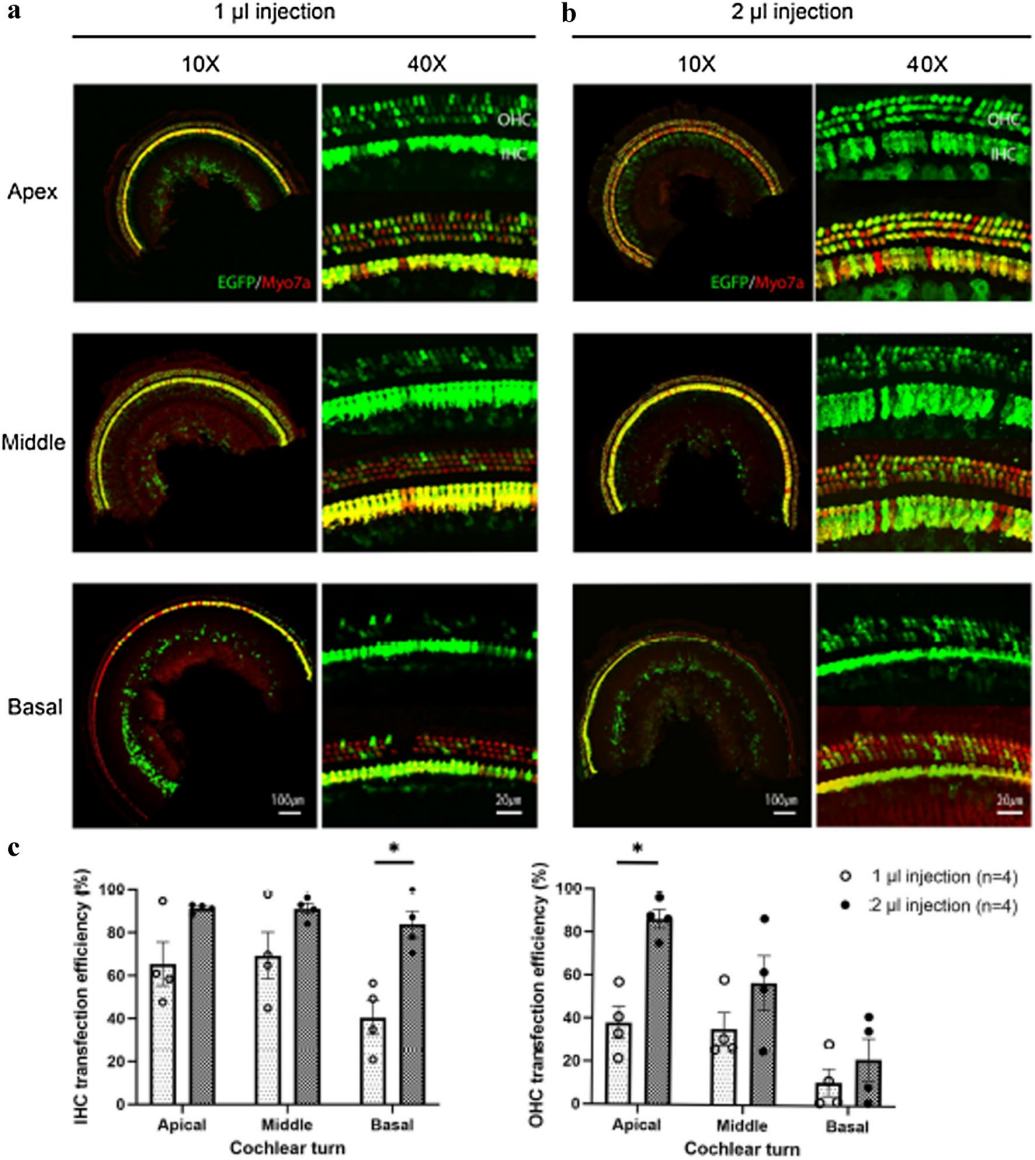
AAV2.7m8 is capable of transducing IHCs and OHCs in the adult mouse cochlea, and the transduction efficiency increased in a dose-dependent manner. (**a**, **b**) Representative 10 × and 40 × images from apical, middle, and basal turns of the cochlea of mice injected with 1 µl (**a**) and 2 µl (**b**) of AAV2.7m8-GFP via the PSC approach. The IHC and OHC transduction rates are better in the animal that received 2 µl of AAV2.7m8-GFP gene delivery, as evidenced by higher number of IHCs and OHCs with GFP expression. (**c**) Quantitative comparison of transduction efficiency of IHCs and OHCs in adult mice that received 1 µl and 2 µl injections revealed a dose-dependent increase in hair cell transduction efficiency, especially in IHCs of the basal turn and OHCs of the apical turn (*p* < 0.05; *t* test). GFP expression is shown in green and Myo7a expression is shown in red. Scale bar represents 100 μm and 20 μm in 10X images and 40X images, respectively. For each animal, hair cell transduction rate was quantified at six different locations along the cochlea: two in the apical turn, two in the middle turn, and two in the basal turn. Data are represented as mean ± SEM). Statistical significance between groups is shown above error bars (*represents *p* < 0.05; *t* test).

We previously showed that AAV2.7m8 is a powerful viral vector for gene delivery in the neonatal mouse inner ear^18^. However, it has been shown that AAV transduction efficiency can be different between neonatal and adult mouse inner ears^16,20,22^. Therefore, we assessed the transduction pattern and efficiency of AAV2.7m8 in the adult mouse inner ear using our newly refined PSC approach. When 1 µl of AAV2.7m8 was delivered via PSC approach by 72 injections of 13.8 nl per injection every 10 s, IHC and OHC transduction rates were 65.3 ± 10.1 and 37.9 ± 7.4% in the apical turn, 69.2 ± 10.8 and 35.2 ± 7.7% in the middle turn, and 40.3 ± 7.8 and 10.8 ± 6.3% in the basal turn of the cochlea (Fig. 5a). Even though the overall transduction rate is lower compared to our previous study in neonatal mouse inner ears, there were some adult mice that had very high rates of IHC and OHC transduction, comparable to neonatal ears. The reduction in overall IHC and OHC transduction rates in the adult mouse inner ear is likely due to the increased technical challenge with adult mouse inner ear gene delivery compared to neonatal ears.

To determine whether overall injection volume affects transduction efficiency, we injected 4 mice with ~ 2 µl of AAV2.7m8-GFP using 72 injections of 27.6 nl per injection every 10 s (Fig. 5b). IHC and OHC transduction efficiencies with 2 µl were 91.2 ± 0.9% and 86.3 ± 4.3% in the apical turn, 91.0 ± 2.5% and 56.9 ± 12.7% in the middle turn, and 83.7 ± 6.3% and 21.6 ± 9.8% in the basal turn of the cochlea (Fig. 5c). When compared with 1 µl injection, the overall transduction rate was higher across the cochlear turns, and the difference in transduction rate was significant in the basal turn of the cochlea for IHCs, and the apical turn of the cochlea for OHCs. This indicates that AAV2.7m8 is capable of transducing cochlear IHCs and OHCs at high levels, and the transduction efficiency in adult mice increases in a dose-dependent manner.

### Hearing is preserved in adult mouse inner ear after 2 µl injection using the PSC approach

**Figure 6:**
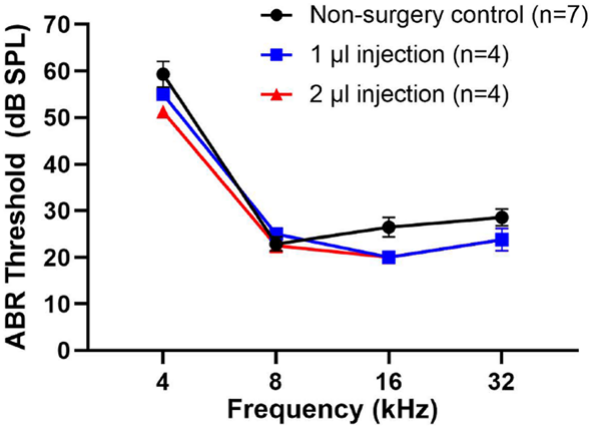
Adult mice injected with 2 µl of AAV2.7m8-GFP did not have hearing loss. ABR threshold elevation was not observed in adult mice at 1 week following 1 µl and 2 µl of AAV2.7m8-GFP injection, compared with non-surgery control mice. Data are represented as mean ± SEM. n represents the number of animals tested.

Even though AAV2.7m8 is capable of transducing cochlear IHCs and OHCs at high levels in the adult mouse inner ear, we had to increase the total injection volume to 2 µl in order to match the transduction efficiency seen in the neonatal mice. Since the adult mouse inner ear is more vulnerable to surgical manipulation and injection volume, we assessed whether a 2 µl injection would have any effect on auditory function in these animals. We found that neither 1 µl nor 2 µl injection volume caused significant ABR threshold shift compared to non-surgery control mice, when the injection parameters were kept below 27.6 nl every 10 s (*p* = 0.77 and 0.58, respectively, one-way ANOVA with post-hoc Scheffe comparison). We also found that the average ABR threshold was not significantly different between mice injected with 1 µl and 2 µl (*p* = 0.96, one-way ANOVA with post-hoc Scheffe comparison, Fig. 6). These results demonstrate that up to 2 µl of fluid volume can be safely injected into the adult mouse inner ear using the PSC approach without causing any significant ABR threshold elevation.

## Discussion

Inner ear gene therapy has been shown to be effective at improving the auditory function of several mouse models of hereditary hearing loss^2,23–28^. While these proof-of-concept studies are very promising, most of these studies require gene delivery to be performed at the neonatal age (before P5), when the mouse inner ear is still not fully mature. In contrast, the human inner ear begins to have auditory perception by ~ 19-week gestation and is fully developed at birth^29^. Therefore, while there are many factors which will determine the success of translating inner ear gene therapy from animal models to humans, it is likely that one must demonstrate effective gene delivery in the mature mammalian inner ear in animal models before successfully implementing gene therapy in human ears. While PSC approach is a well-established surgical approach for gene delivery in neonatal mouse inner ear, few studies have examined the impact of PSC approach on hearing outcome in the adult mouse inner ear^14,16,20^. Even though the anatomy between the neonatal and adult mouse inner ear is similar, one major difference is the fact that the adult otic capsule is completely ossified, whereas the neonatal otic capsule is still cartilaginous^4,19^. This difference makes accessing the adult inner ear much more challenging, and potentially more traumatic. Indeed, we found that adult mouse inner ear is more susceptible to hearing loss after PSC gene delivery compared to neonatal ears^18^. Therefore, we decided to see if we could refine the surgical techniques of PSC gene delivery in the adult mouse inner ear to minimize hearing loss. We examined the three main surgical steps involved with PSC gene delivery: (1) fenestration of PSC, (2) insertion of injection tubing into the PSC, and (3) injection of fluid into the PSC. We found that prolonged perilymphatic leakage and injection rate have significant effects on hearing in the adult mouse inner ear.

Fluid leakage from the fenestra is a good indicator of having obtained access to the perilymphatic space during canalostomy approach. In order to decrease the fluid volume and pressure in the perilymphatic space to accommodate for gene therapy injection, Suzuki et al. recommended waiting for 5 min after the fenestra of the PSC wall is opened to allow perilymph to leak out^20^. Similarly, in a study by Yoshimura et al., the authors recommended fenestrating the PSC when performing round window gene delivery to allow perilymphatic leakage in order to decrease the pressure in the inner ear^13^. Recently, a study reported that single and dual AAV2 injections using the combined round window injection and PSC fenestration have high transduction efficiency to both IHCs and OHCs^30^. In the present study, we found that a major factor for hearing loss in adult mice undergoing PSC gene delivery is perilymphatic leakage. We found that mice with a fenestra opening time of 10 min prior to securing the injection tubing exhibited significantly higher ABR thresholds compared to non-surgery control mice (Fig. 2b). The concept of minimizing the duration of time for perilymphatic leakage is well known in the otologic surgery literature. In cholesteatoma surgery, when the cholesteatoma has eroded through the otic capsule (most commonly involving the horizontal semicircular canal), it is generally recommended to leave the cholesteatoma matrix on the perilymphatic fistula to avoid perilymphatic leakage and exposure of the inner ear^31,32^. In cases where a decision is made to open the eroded horizontal semicircular canal wall, it is usually recommended to seal the fistula immediately in order to minimize perilymphatic leakage and preserve inner ear function^33,34^. Therefore, our data suggest that it is important to minimize the leakage of perilymph after the fenestra on the posterior semicircular canal is opened in order to minimize hearing loss. We recommend inserting and securing the injection tubing as soon as the fenestra on the posterior semicircular canal is opened.

The delivery of fluid volume into the inner ear could potentially cause barotrauma and mechanical trauma to the inner ear. In mice injected with 1 µl of viral vectors via multiple small doses 10 s apart, a larger volume of fluid per dose was significantly associated with ABR threshold shift (Fig. 4d). In addition, mice that received fast injection rate (20 injections in 30 s) exhibited significant hair cell loss (Fig. 4b,c) and ABR threshold elevation (Fig. 4a). Both large fluid volume per injection and shortened duration between injections can induce large and fast displacement of perilymph, which may lead to increased pressure within the cochlea, causing mechanical damage. Severe IHC and OHC loss observed in the basal turn of the cochlea compared to apical turn of the cochlea (Fig. 4b) may reflect the increased susceptibility of hair cells at the cochlear base to trauma^35^. Clinical experience with hearing preservation cochlear implant surgery also supports our findings. Thick diameter of cochlear implant array is analogous to large volume per injection in this study. It has been shown that larger diameter cochlear implant array leads to higher insertion force and results in increased risk of loss of residual hearing during cochlear implant surgery^36^. Similarly, an increase in implant insertion speed is analogous to injection speed (the time interval between injections) in the present study. It has also been shown that increased cochlear implant insertion speed can cause adverse effects on residual hearing during cochlear implant surgery^37,38^. Therefore, our data suggest that a smaller volume of fluid per injection with slower injection speed (longer time interval between injections) offers the best chance for hearing preservation in the adult mouse inner ear with PSC gene delivery.

In our previous study, we showed that AAV2.7m8 is capable of transducing neonatal cochlear IHCs and OHCs at high levels^18^. In this study, we tested the transduction efficiency of AAV2.7m8 in the adult mouse cochlea. We found that AAV2.7m8 was also capable of transducing the adult cochlear IHCs and OHCs, but the overall transduction rate was lower than what we observed in the neonatal inner ears^18^. The decrease in transduction efficiency in the adult mouse inner ear has been reported in other studies^9,39,40^. In a study comparing the transduction efficiency of several AAV serotypes between neonatal and adult mouse inner ears, Shu et al. found that the viral transduction efficiency in the adult mouse inner ear was significantly lower than the neonatal mouse inner ear^9^. In another study, when exogenous *Tmc1* gene was delivered into the inner ear of *Tmc1* deficient mice using Anc80L65, infected hair cell rates decreased as a function of injection age from 93% at P1 to 3% at P14^40^. A recent study in which AAV9-PHP.B was used as the viral vector for inner ear gene delivery in a mouse model of Usher syndrome type 3A, the authors observed that AAV9-PHP.B was able to transduce both OHCs and IHCs in neonate mice (P0-P1), while adult mice (P28) exhibited transduction in only IHCs^39^. Although this decrease may be due to the maturation of cellular architecture of the inner ear that prevents the diffusion of AAV to infect hair cell^9^, the exact mechanism involved is still unclear. Therefore, further study is needed to better understand the mechanism behind this phenomenon.

We observed that the transduction rates of IHCs and OHCs increased in a dose-dependent manner with AAV2.7m8, with no significant ABR threshold shift up to a total injection volume of 2 µl which was the highest dose tested in this study. The average volume of perilymphatic space in mice is 0.62–1.72 µl^41,42^. Therefore, it is interesting that an injection volume of 2 µl did not negatively impact auditory function. In addition to our results, other studies have also shown that up to 2 µl of fluid volume can be delivered into the mouse inner ear without any significant effect on the auditory function^14,43–45^. The ability of the mouse inner ear to accommodate a large fluid volume may be explained by the presence of a relatively large and patent cochlear aqueduct, which communicates between the perilymphatic and subarachnoid spaces^18,46^. The cochlear aqueduct essentially acts as an outflow valve which allows excess fluid volume to escape into the subarachnoid space. The fact that up to 2 µl of fluid volume can be safely delivered to the adult mouse inner ear is particularly useful in gene therapy studies requiring the use of multiple viral vectors (e.g. dual-AAV approaches for delivering large cDNA), since these studies often require larger injection volume.

The posterior semicircular canal can be safely accessed in humans by performing a cortical mastoidectomy. It is typically used in patients who undergo posterior semicircular canal plugging procedure for intractable vertigo as a result of benign paroxysmal positional vertigo (BPPV). According to a study examining the surgical outcomes in 61 patients with intractable BPPV, the rate of vertigo resolution was 100%, and complication rate such as hearing loss was extremely low ^47^. Therefore, we believe that the PSC approach can be used for inner ear gene delivery in human ears. However, even though we found that the PSC is the easiest surgical approach for inner ear gene delivery in mouse ears, other surgical approaches are likely to be easier for inner ear gene delivery in human ears. This is due to the anatomical differences between the mouse and human ears. For example, the PSC is the most prominent and easily accessed semicircular canal in mice, but the horizontal semicircular canal is the most prominent and easily accessed semicircular canal in humans. In addition, in order to access the round window in mice, the tympanic bulla needs to be opened, whereas in humans, the round window can be accessed directly through the ear canal, without performing a cortical mastoidectomy. Therefore, it is important to keep these anatomical differences in mind when comparing the surgical approaches for inner ear gene delivery between mouse and human ears.

In conclusion, our study shows that successful gene delivery through the PSC in the adult mouse inner ear is feasible, but it requires modifications to the neonatal PSC gene delivery techniques. We found that the auditory function in adult mice can be preserved by reducing the PSC opening time to minimize perilymphatic leakage, as well as utilizing a slower fluid injection rate. In addition, the synthetic AAV2.7m8 is capable of transducing adult mouse IHCs and OHCs with high efficiency. It is our hope that the detailed methods described in this study can be utilized for safe and efficient gene delivery in the adult mouse inner ear.

## Methods

### AAV vector construction

The AAV2.7m8-CAG-eGFP (9.75 × 10^12^ GC/mL) was produced by the Research Vector Core at the Center for Advanced Retinal and Ocular Therapeutics (University of Pennsylvania). The virus consists of a CAG promoter derived from InvivoGen pDRIVE CAG plasmid (InvivoGen, San Diego, CA), the cDNA encoding enhanced GFP (eGFP) protein, and the bovine growth hormone (bGH) polyadenylation signal. The virus was manufactured after triple transfection of HEK293 cells and was isolated and purified by microfluidization, filtration, cation exchange chromatography, density gradient ultracentrifugation, and diafiltration in PBS. The purified virus was passed through a 0.22-μm filter using a sterile 60-ml syringe and syringe filtered, and stored frozen at − 80 °C in sterile tubes until use.

### Animal surgery

Animal surgery was approved by the Animal Care and Use Committee at the National Institute on Deafness and Other Communication Disorders (NIDCD ASP1378-18). The study was done in compliance with ARRIVE guidelines. All animal procedures were done in compliance with the ethical guidelines and regulations set forth by the Animal Care and Use Committee at NIDCD. Adult (P30-90) CBA/J mice were used in this study. Anesthesia was induced using isoflurane gas (Baxter, Deerfield, IL) through a nose cone at a flow rate of 0.5 L/min. Gene delivery was done using the PSC approach. A post-auricular incision was made using small scissors. The soft tissues were bluntly dissected to expose the PSC. The facial nerve was identified after dividing the sternocleidomastoid muscle. In order to locate the PSC, the facial nerve was followed superiorly and posteriorly. The PSC could be found about 3–4 mm posteriorly from the ear canal. To expose lumen of the ossified PSC, a 27-guage hypodermic needle was used. Perilymphatic leakage was used as the confirmation for successful access to the PSC lumen. A Nanoliter Microinjection System (Nanoliter2000, World Precision Instruments, Sarasota, FL) was used in conjunction with a polyethylene tube (0.1222 mm diameter, MicroLumen, Oldsmar, FL) attached with glass micropipette (1.0 mm outer diameter, 0.75 mm inner diameter, Shutter instruments, Novato, CA) to load viral vector. The polyethylene tube was inserted into the PSC lumen. Cyanoacrylate glue was used to secure the injection tubing around the PSC fenestration in order to prevent perilymphatic leakage from the injection site. Perilymphatic leakage is carefully observed under the operating microscope during the injection process. AAV2.7m8-CAG-EGFP (9.75 × 10^12^ GC/mL) was injected according to following different volume and rates to define best option for fluid injection: 13.8 nl × 72 injections every 10 s (total volume 993.6 nl), 27.6 nl × 36 injections every 10 s (total volume 993.6 nl), 46 nl × 20 injections every 10 s (total volume 920 nl), 46 nl × 20 injections in 30 s total (total volume 920 nl), and 27.6 nl × 72 injections every 10 s (total volume 1.987 µl). Incision was closed with 5–0 vicryl sutures.

### Auditory brainstem response

Auditory brainstem response (ABR) testing was performed to evaluate hearing sensitivity 7–10 days after surgery. Animals were anesthetized with ketamine (100 mg/kg) and dexmedetomidine (0.375 mg/kg) via intraperitoneal injections and placed on a warming pad inside a sound booth (ETS-Lindgren Acoustic Systems, Cedar Park, TX). The animal’s temperature was maintained using a closed feedback loop and monitored using a rectal probe (CWE Incorporated, TC-1000, Ardmore, PN). Sub-dermal needle electrodes were inserted at the vertex (+) and test-ear mastoid (−) with a ground electrode under the contralateral ear. Stimulus generation and ABR recordings were completed using Tucker Davis Technologies hardware (RZ6 Multi I/O Processor, Tucker-Davis Technologies, Gainesville, FL, USA) and software (BioSigRx, v.5.1). The auditory stimuli were presented in a closed-field configuration using an MF-1 speaker (Tucker-Davis Technologies) coupled to the test ear via a 2 cm tube and modified 10 μl pipette tip. Click and tone-burst ABR thresholds were measured at 4, 8, 16, and 32 kHz using 3-ms, Blackman-gated tone pips presented at 29.9/sec with alternating stimulus polarity. At each stimulus level, 512–1024 responses were averaged. Thresholds were determined by visual inspection of the waveforms and were defined as the lowest stimulus level at which any wave could be reliably detected. A minimum of two waveforms was obtained at the threshold level to ensure repeatability of the response. Physiological results were analyzed for individual frequencies, and then averaged for each of these frequencies from 4 to 32 kHz.

### Immunohistochemistry and quantification

After completion of functional testing, mice were euthanized by CO2 asphyxiation followed by decapitation. Temporal bones were harvested and fixed overnight with 4% paraformaldehyde followed by decalcification in 120 mM EDTA for 4 days. The vestibular organs and cochlear sensory epithelia were micro-dissected, blocked, and labeled with rabbit anti-myosin 7a antibody to label hair cells (1:200, product # 25–6790, Proteus BioSciences, Ramona, CA), and chicken anti-GFP antibody to label GFP (1:1000, product # ab13970, abcam, Cambridge, MA), and Hoechst stain (1:500, product # 62,249, Life Technologies, Carlsbad, CA) to label nuclei. Primary and secondary antibodies were diluted in PBS. Images were obtained using a Zeiss LSM780 confocal microscope at 10 × and 40 × using z-stacks.

For quantification of cochlear hair cell and supporting cell transduction rate, two 40 × images were taken at the apex, middle turn, and base of cochlea. The number of hair cells and supporting cells with GFP expression was counted and averaged at each location along the cochlea. Each 40 × image contains ~ 30 IHCs and ~ 90 OHCs. The overall transduction rate was calculated by averaging the transduction rates obtained from the entire cochlea. For quantification of utricular hair cell transduction rate, two 40 × images (each containing ~ 300 vestibular hair cells) were taken per utricle specimen and the number of hair cells with GFP expression was counted and averaged.

### Statistics

Student’s *t* test (two-tailed) was used to assess differences in transduction efficiency. It has been shown that different AAV serotypes can have different transduction efficiencies in different regions of the cochlea^45^. Therefore, transduction efficiencies from each region of the cochlea (apex, middle turn, and cochlear base) were treated as separate measurements in the calculation of mean, standard error, and statistical significance. For ABR threshold, one-way analysis of variance (ANOVA) was used to assess differences in the thresholds. Post-hoc analysis was performed using the Scheffe method. The *p* value of < 0.05 indicates statistical significance.

## Supplementary Information

Supplementary Information.

## Data Availability

The datasets generated during and/or analyzed during the current study are available from the corresponding author on reasonable request.
