# Supplementary material for: Refining surgical techniques for efficient posterior semicircular canal gene delivery in the adult mammalian inner ear with minimal hearing loss
Source: Sci Rep. 2021 Sep 22;11:18856. doi: 10.1038/s41598-021-98412-y (PMC8458342; doi:10.1038/s41598-021-98412-y)
Supplement: Supplementary file 1 — Supplementary Information. [file 41598_2021_98412_MOESM1_ESM.docx]

**Supplementary Figure 1**


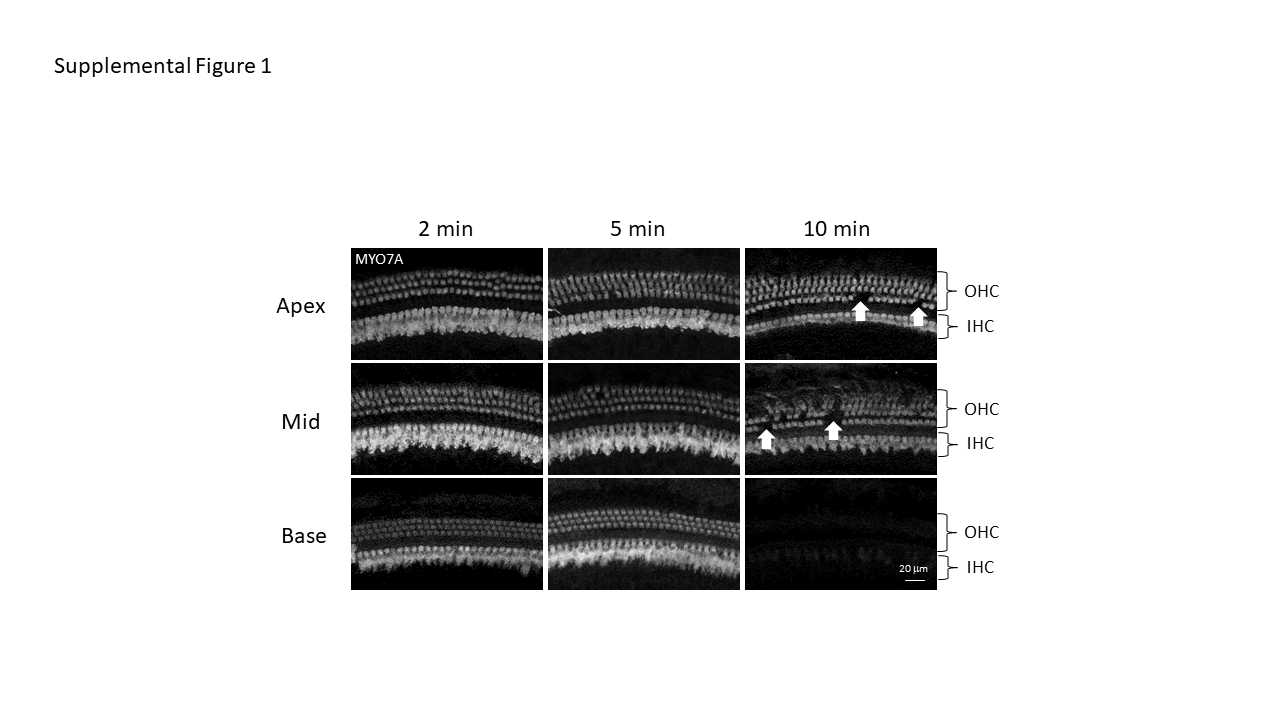


Supplemental Figure 1: Prolonged perilymphatic leakage causes hair cell loss. Confocal images of the cochlea in mice that underwent PSC fenestration for 2 minutes, 5 minutes, and 10 minutes are shown. Hair cells are labeled using anti-MYO7A antibody (white). No obvious HC loss is observed in animals in the 2-minute and 5-minute PSC fenestration groups , but significant IHC and OHC loss is seen in animals in the 10-minute PSC fenestration group (white arrows). In fact, complete IHC and OHC loss is seen at the cochlear base. Scale bar: 20 μm.
